# Scalable and sustainable process of spike spherical Mg(OH)_2_ adsorbent from magnesite by ammonia-cycle method for dye removal

**DOI:** 10.1371/journal.pone.0332393

**Published:** 2025-09-16

**Authors:** Xiumei Duan, Chao Wang, Yue Zhang, Xiuyuan Zuo, Hai Liang, Xibao Zhang

**Affiliations:** 1 Liaoning Provincial Engineering Research Center for High-Value Utilization of Magnesite, Liaoning Key Laboratory of Chemical Additive Synthesis and Separation, Yingkou Institute of Technology, Yingkou, China; 2 Key Laboratory of Industrial Ecology and Environmental Engineering (Ministry of Education, China), School of Environmental Science and Technology, Dalian University of Technology, Dalian, China; 3 Hebei University of Environmental Engineering, Qinhuangdao, Hebei, China; 4 Liaoning Hongcheng Environmental Protection Co., Ltd. Yingkou East Branch, Yingkou, China; Qatar University, QATAR

## Abstract

This study presents an innovative, and environmentally friendly synthesis process for spike spherical magnesium hydroxide (SSMH) using magnesite and an ammonia-cycle method, which eliminates waste liquid, gas, and chemical reagent pollution. The process involves calcining magnesite to obtain calcined magnesite, which reacts with (NH_4_)_2_SO_4_ to produce magnesium sulfate and ammonia. Subsequently, magnesium sulfate reacts with ammonia water to generate SSMH. The optimized conditions for the extraction of magnesium ion (Mg²⁺) are as follows: (NH_4_)_2_SO_4_ concentration of 2.4 mol/L, 4 g of calcined magnesite powder, and a reaction time of 1.5 h, resulting in an extraction rate of Mg²⁺ of 93.4%. The optimized conditions for the precipitation of Mg² ⁺ are as follows: Mg² ⁺ concentration of 0.7–1.3 mol/L, NH₃H₂O/Mg² ⁺ molar ratio of 9, reaction temperature of 60°C, and reaction time of 1 h, with a precipitation rate of Mg²⁺ of about 85%. After five ammonia cycles, the precipitation rate of Mg² ⁺ stabilizes at 85%. Scanning Electron Microscopy (SEM) confirms the spike spherical structure of the SSMH, with uniform particle diameters and a particle size of 2 μm. Adsorption studies indicate a maximum adsorption capacity (Q_m_) of 150.4944 mg/g for Reactive Red X-3B (RRX) dye at 25°C, fitting well with the Langmuir and pseudo-second-order kinetic model. The adsorption is primarily chemisorption-driven. In conclusion, the ammonia-cycle method for SSMH from magnesite is an environmentally friendly and sustainable approach. SSMH with its high adsorption capacity and spike spherical structure, is effective for treating RRX aqueous solution and has potential for broader applications in removing heavy metals, persistent organic compounds, nitrogen, and phosphorus from various polluted sources.

## 1 Introduction

Dyes are important chemical products, with an annual global production reaching several million tons. As the global economy continues to grow, the demand for dyes increases year by year. Dyes are widely used in various industries, such as textiles, papermaking, printing, plastics, and food [1]. The textile and dyeing industries are the main application areas for dyes, accounting for over 70% of global dye consumption. However, during the production and application of dyes, a large amount of wastewater containing complex chemical components is generated. This wastewater contains a large number of unreacted or degraded dye molecules, which significantly increase the chemical oxygen demand (COD) and biological oxygen demand (BOD) of the wastewater, severely affecting the ecological environment of water bodies [2].

Multiple approaches, including adsorption, membrane separation, precipitation, oxidation, catalytic oxidation, coagulation, and aerobic, anaerobic biological treatments [3], have been developed to remove or recycle dye from wastewater. Compared with other method, adsorption emerges as a promising approach for remove dyes due to its high efficiency, rapid capture rate, operational simplicity, and cost-effectiveness. Various versatile adsorbents such as zeolite, biochar, clay, chitosan, graphene, graphene oxide and metal oxides, and hydroxides have been developed for removing various different types of reactive red dyes from aquatic ecosystem [4–11]. Among them, magnesium hydroxide, as a magnesium-based material produced from magnesite or seawater possesses outstanding characteristics, including porous structure, abundant functional groups, high chemical stability, and tunable surface, positioning it as a promising adsorbent [12–18]. Jumaeri et al [14] conducted a study on the removal of Congo red from aqueous solutions using magnesium hydroxide extracted from seawater bittern as an adsorbent. Under the conditions of pH 8 and a contact time of 90 minutes, with an initial Congo red concentration of 29 mg/L, the adsorption capacity was 46.3 mg/g. Jiang et al [15] prepared novel sunflower torus-like magnesium hydroxide microsphere particles using a self-assembly method. Sunflower torus-like magnesium hydroxide is an effective adsorbent for dye removal. Under optimized conditions, the removal rates of reactive blue 19 and alizarin red S were 91.65% and 83.03%, respectively. The maximum adsorption capacities of the microspheres for alizarin red S and reactive blue 19 were 349.85 mg/g and 231.78 mg/g at 25 °C, respectively. Reactive blue 19 and alizarin red S were adsorbed on the surface of sunflower torus-like magnesium hydroxide through the formation of hydrogen bonds. Magnesium hydroxide is currently limited in its applications due to the large amount of reagents required during synthesis, ammonia pollution, water pollution, as well as its low specific surface area and difficulties in filtration [15,19]. Therefore, it is necessary to adopt new processes to prepare large-particle magnesium hydroxide adsorbent particles with porous structures in order to address these issues.

To address these challenges, This study aims to:(1) propose an environmentally friendly, scalable and sustainable process for producing SSMH adsorbents from magnesite via an ammonia-cycle route; (2) comprehensively evaluate SSMH’s performance, establish adsorption isotherm models and adsorption kinetics models, and correlate these with morphological and other physicochemical analyses to clarify the adsorption behavior and mechanism of SSMH toward RRX dyes, thereby providing a solid basis for practical application.

## 2 Materials and methods

### 2.1 Raw materials

Magnesite was obtained from Dashi Bridge City, Liaoning Province, China. XRF analysis revealed its composition as follows: MgO 42.7%, CaO 55.2%, Fe₂O₃ 0.45%, SiO₂ 0.66%, Al₂O₃ 0.28%, and other components 0.71%.

The chemical reagents (NH_4_)_2_SO_4_, ammonia water, sodium chloride, hydrochloric acid, sodium hydroxide, Reactive Red X-3B (RRX) were used in the present work. RRX was purchased from Tianjin Baima. The remaining chemical reagents were purchased from Tianjin Zhiyuan. All reagents were analytically pure (A.R. grade).

### 2.2 SSMH synthesis

#### 2.2.1 The synthesis process.

**Fig 1:**
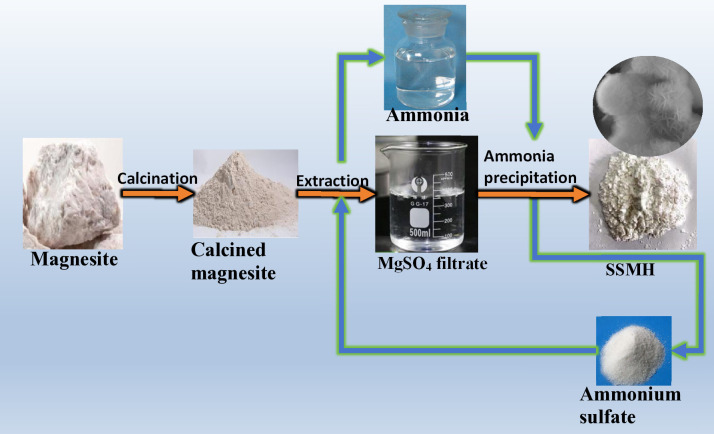
SSMH synthesis process by ammonia-cycle method.

The synthesis of SSMH was illustrated in Fig 1. First, the magnesite was crushed by a heavy hammer, passed through a 200-mesh sieve, and the undersize magnesite was calcined and calcined magnesite was obtained. Second, (NH_4_)_2_SO_4_ was reacted with calcined magnesite in a four-neck flask to leach Mg^2+^, NH_3_^.^H_2_O was recovered, simultaneously. Third, NH_3_.H_2_O was reacted with MgSO_4_ to produce SSMH, while (NH_4_)_2_SO_4_ was recovered. The reaction equations for the synthesis of SSMH from magnesite were shown in reactions 1–3.

MgCO_3_ →MgO+CO_2_ (reaction 1)

MgO+(NH_4_)_2_ SO_4_ →MgSO_4_ +2NH_3_^.^H_2_O (reaction 2)

MgSO_4_ +2NH_3_^.^H_2_O→Mg(OH)_2_ +(NH_4_)_2_ SO_4_ (reaction 3)

As shown in Fig 1, using magnesite as the raw material, SSMH was synthesized by the calcination-(NH_4_)_2_SO_4_ leaching-ammonia precipitation method. The by-product ammonia gas generated from the leaching of calcined powder was recovered and used for the magnesium precipitation reaction. The (NH_4_)_2_SO_4_ produced in the magnesium precipitation reaction was also recovered and reused for the leaching of Mg^2+^, forming an ammonia cycle with no ammonia gas pollution. This process reduced the amount of additional chemicals required and lowered the production cost, making it an environmentally friendly synthesis method.

#### 2.2.2 Optimization of conditions for leaching Mg²⁺ from calcined magnesite.

The magnesite was first crushed with a heavy hammer and passed through a 200-mesh sieve. The undersize fraction was then calcined at 800 °C for 1 h [20] in air to obtain calcined magnesite. Subsequently, 4.0000 ± 0.0001 g calcined magnesite was combined with an (NH_4_)_2_SO₄ solution (0.65, 1.31, 2.0, 2.4, 2.8 mol/L) in a four-neck flask, the volume was 50 mL, stirred at 150 rpm, heated (80, 90, 100, 110, 120 °C) for a prescribed period (0.5, 1.0, 1.5, 2.0, 2.5 h), filtered. The residue was washed three times, and the Mg² ⁺ content in solution was estimated by EDTA method and [21] the Mg² ⁺ extraction efficiency was calculated.

#### 2.2.3 Optimization of conditions for the reaction of Mg²⁺ with NH_3_^.^H_2_O to produce SSMH.

The Mg² ⁺ leaching solution was concentrated by evaporation. Ammonia water (NH₃·H₂O/Mg² ⁺ ratios: 6, 7, 8, 9, 10) and Mg² ⁺ leaching solutions (0.1, 0.4, 0.7, 1.0, 1.3 mol/L) were reacted at a certain temperature (30, 40, 50, 60, 70 °C) for a period of time (0.2,0.5,1.0, 2.0,3.0 h). The reaction mixture was then filtered, and the residue on the filter paper was identified as SSMH. The SSMH was washed three times with distilled water. The Mg² ⁺ content in the solution was determined by the EDTA method, and the Mg² ⁺ precipitation efficiency was calculated.

#### 2.2.4 Ammonia recycling experiment.

4.0000 ± 0.0001 g calcined magnesite and 2.4 mol/L (NH₄)₂SO₄ solution was reacted at 100 °C for 1.5 h, yielding a Mg² ⁺ -rich solution and an ammonia distillate. Subsequently, 50 mL of 0.7 mol/L Mg^2+^ solution was reacted with 13.5 mL of ammonia solution (NH₃·H₂O/Mg^2+^ molar ratio: 9) for 1 hour. The reaction mixture was then filtered using a vacuum filter. SSMH was washed three times with distilled water, and the Mg² ⁺ precipitation efficiency was calculated. During the first synthesis of SSMH, (NH_4_)_2_SO_4_ and ammonia solution were purchased from Tianjin Zhiyuan Chemical Reagent Co., Ltd. For the 2nd to 5th syntheses of SSMH, (NH_4_)_2_SO_4_ was entirely sourced from the filtrate of the ammonia synthesis of SSMH, and half of the ammonia solution was derived from the Mg^2+^ leaching distillate, while the other half was purchased from Tianjin Zhiyuan Chemical Reagent Co., Ltd.

### 2.3 The adsorption of RRX dye by SSMH

#### 2.3.1 Optimization of adsorption conditions.

Some SSMH (0.05, 0.10, 0.15, 0.20, 0.25 g) was added to 100 mL of 80 mg L ⁻ ¹ RRX aqueous solution, pH value (3, 5, 7, 9) was adjusted. The mixture was magnetically stirred for a period (5, 10, 20, 30, 40 min). Afterward, it was allowed to settle for a period of time (15, 30, 60, 90, 120 min) to separate the filtrate from the precipitate. The RRX concentration in the supernatant was measured and the removal efficiency was calculated. Each experimental condition was repeated three times.

#### 2.3.2 Adsorption isotherms.

The adsorption capacity (Q_e_, mg/g) was based on isothermal equilibrium experiments studies, which were carried out under 25°Cfor 3 h. Moreover, the initial RRX dye concentration was 60–160 mg/L, and the RRX dye solution volume was 100 mL, and 0.1 ± 0.0001 g SSMH was added to every beaker. The adsorption capacities were then calculated using Equation 1(Eq. (1)).


$$Q_{e}=\frac{C_{0}-C_{e}}{m}\times V$$ (1)

Q_*e*_ (mg/g): the equilibrium adsorption amount; C_0_ (mg/L): initial concentration; C_e_ (mg/L): adsorption equilibrium concentration; m (g): adsorbent mass; V (L): RRX aqueous solution volume.

The Langmuir equation (Eq. (2)) and Freundlich equation (Eq. (3)) were employed to assess the isothermal adsorption performance.


$$Q_{e}=\frac{Q_{m}C_{e}K_{L}}{1+K_{L}C_{e}}$$ (2)

$$\;Q_{e}=K_{F}C_{e}^{1/n}\;$$ (3)

K_L_ (L/mg): the Langmuir isotherm adsorption rate constant; K_F_: the Freundlich isotherm adsorption constant; C_e_ (mg/L): adsorption equilibrium concentration; Q_*e*_ (mg/g): the equilibrium adsorption amount; Q_m_ (mg/g): the maximum adsorption capacity for a monolayer; n: the Freundlich isotherm characteristic constant.

#### 2.3.3 Adsorption kinetics.

The removal rates for the treatment of RRX dye solution with SSMH microsphere particles were investigated by the preceding kinetic model. The initial concentration was 80 mg/L, the volume was 100 mL, the mass of SSMH was 0.1 ± 0.0001 g, and the contact time was implemented for 0–180 min at 25 °C, The adsorption kinetics experiments were evaluated by the pseudo-first-order kinetic equation (Eq. (4)) and pseudo-second-order kinetic equation (Eq. (5)) in this work.


$$\text{log}(Q_{e}-Q_{t})=\text{log}Q_{e}-\frac{K_{1}t}{2.303}$$ (4)

$$\frac{t}{Q_{t}}=\frac{t}{Q_{e}}+\frac{1}{{K_{2}Q}_{e}^{2}}$$ (5)

Q_e_(mg/g): the adsorption amount at equilibrium; Q_t_ (mg/g): the adsorption amount at time t; K_1_ (min^-1^): the first-order kinetic parameter; K_2_ (g/mg/min): the second-order kinetic parameter.

The RRX dye concentrations (C_t_, mg/L) were determined by spectrophotometry after adsorption for a period of time. Eq. (6) was used to evaluate the adsorption rate (*N*_t_). Eq. (7) was also adopted to estimate the adsorption capacity (Q_t_).


$$N_{t}=\frac{C_{0}-C_{t}}{C_{0}}\times 100\%$$ (6)

$$Q_{t}=\frac{C_{0}-C_{t}}{m}\times V$$ (7)

*N*_t_: the removal rate at any time (t (min)) during the adsorption process; C_0_ (mg/L): initial concentration; C_t_ (mg/L): the concentration values of adsorbed substances after the adsorption time of t (min); m (g): SSMH adsorbent mass; V (L): the solution volume.

### 2.4 Characterization of SSMH and analytical methods

The morphologies and structures of the samples were examined by SEM (Regulus 8100, Hitachi, Japan). Specific surface area was evaluated by the brunauer-emmett-teller method (ASAP 2460, Micromeritics, USA). Solution pH was measured with a pHS-3D pH meter. The concentration of RRX dye was determined by UV–visible spectrophotometry (UV-3600i Plus, Shimadzu, Japan). The Mg² ⁺ concentration in the solution was determined by the EDTA titrimetric method.

## 3 Results and discussion

### 3.1 Optimization of Mg^2+^ extraction conditions

**Fig 2:**
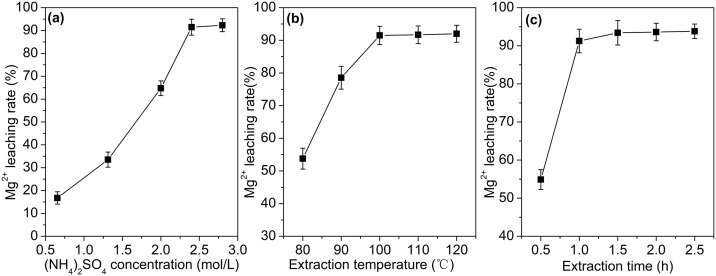
Optimization of leaching conditions for Mg^2^^+^ from calcined magnesite. (a)Effect of (NH_4_)_2_SO_4_ concentration; (b)Effect of temperature;(c)Effect of reaction time.

The effects of (NH_4_)_2_SO_4_ concentration, temperature and time on the extraction rate of Mg^2+^ are shown in Fig 2.

The effect of (NH_4_)_2_SO_4_ concentration on the leaching rate of Mg² ⁺ is shown in Fig 2a. The experiment was conducted using 4.0000 ± 0.0001 g of calcined magnesite and 50 mL (NH_4_)_2_SO_4_ solution, with ammonia vaporization carried out at 100°C for 1 hour. As the (NH_4_)_2_SO_4_ concentration increases, the leaching rate of Mg² ⁺ also increases. When the (NH_4_)_2_SO_4_ concentration is 0.65 mol/L, the leaching rate of Mg² ⁺ is only 16.76%. With further increase in the (NH_4_)_2_SO_4_ concentration, the leaching rate of Mg² ⁺ increases linearly. When the (NH_4_)_2_SO_4_ concentration reaches 2.4–2.8 mol/L, the leaching rate of Mg² ⁺ exceeds 90%. At low concentrations, increasing the concentration of (NH_4_)_2_SO_4_ provides more reactants, which promotes the leaching of Mg² ⁺ . However, once the concentration reaches a certain level, the reactants are already sufficient, and further increasing the concentration has limited effect on improving the leaching rate.

The effect of reaction temperature on the leaching rate of Mg² ⁺ is shown in Fig 2b. The experiment was conducted with 50 mL of 2.4 mol/L (NH_4_)_2_SO_4_ solution and 4.0000 ± 0.0001 g of calcined magnesite, and ammonia was vaporized for 1 hour. As the temperature continues to rise, the leaching rate of Mg^2+^ also increases accordingly. However, when the reaction temperature reaches 100 °C or above, this trend becomes relatively stable. Therefore, the optimal temperature is determined to be 100 °C. An increase in temperature can enhance the thermal motion of reactant molecules, thereby accelerating the reaction rate and consequently improving the leaching efficiency. However, when the temperature becomes excessively high, the reaction is already quite complete, and further elevation of temperature has limited effect on boosting the leaching rate [19].

50 mL of (NH_4_)_2_SO_4_ solution and 4.0000 ± 0.0001 g of calcined magnesite was reacted at 100°C. The effect of reaction time on the leaching rate of Mg² ⁺ is shown in Fig 2c. As the reaction time increases, the extraction rate of Mg^2+^ gradually increases. When the reaction time reaches 1.5 hours, the extraction rate curve begins to stabilize. Therefore, the evaporation time is determined to be 1.5 hours. In the early stages of the reaction, extending the leaching time can promote the progress of the reaction and increase the leaching rate. However, once the reaction has proceeded to a certain extent, the leachable magnesium has been mostly extracted, and further extending the leaching time has limited effect on improving the leaching rate.

### 3.2 Optimization of the synthesis conditions for SSMH

**Fig 3:**
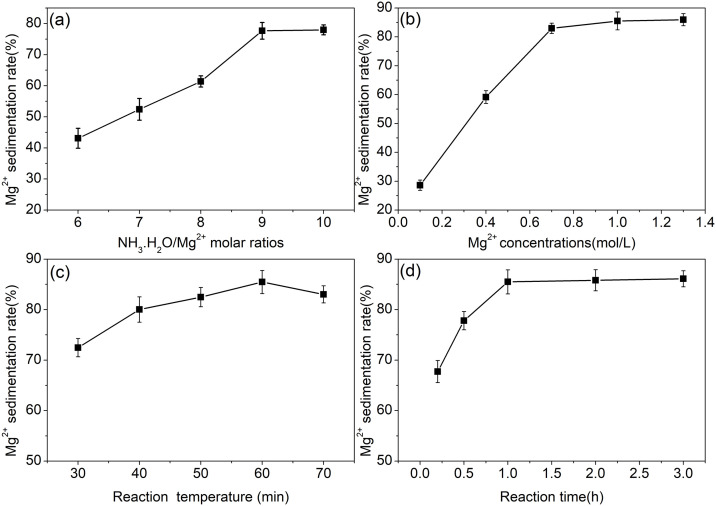
Optimization of Mg^2^^+^ precipitation with NH_3_H_2_O. (a)Effect of ammonia dosage on the precipitation efficiency of Mg² ⁺ ; (b)Effect of Mg² ⁺ concentration; (c)Effect of reaction temperature; (d)Effect of reaction time.

The effects of molar ratios of NH_3_H_2_O/Mg^2+^, Mg^2+^ concentrations, reaction temperature and reaction time on Mg^2+^ precipitation rate were shown in Fig 3.

50 mL of Mg^2+^ leaching solution was divided into 5 portions, and the Mg^2+^ concentration was adjusted to 0.35 mol/L. Ammonia water was added in volumes of 7.9, 9.2, 10.5, 11.9, and 13.2 mL, respectively, to the round-bottom flasks. The reaction was carried out at 60 °C for 1 h. After that, the treated solutions were vacuum filtered, washed, and the Mg^2+^ concentration was measured to calculate the precipitation rate of Mg^2+^. The results are shown in Fig 3a. When the volume of ammonia water added was below 11.9 mL (NH_3_H_2_O/Mg^2+^ molar ratio:9), the precipitation rate of Mg^2+^ increased significantly with the increase in ammonia volume. When the volume increased to 11.9 mL, the recovery rate rose to 77.67%. Further increasing the volume to 13.2 mL (NH_3_H_2_O/Mg^2+^ molar ratio:10) led to a recovery rate of 77.98%. The magnesium precipitation rate remained essentially unchanged between NH_3_H_2_O/Mg^2+^ molar ratio of 9–10. Therefore, an ammonia water addition volume of 11.9 mL was selected. According to the chemical reaction equation, when NH_3_H_2_O/Mg^2+^ molar ratio is 2:1, the added volume should be 2.6 mL. However, since ammonia water is a weak electrolyte and partially ionizes in water, the precipitation of Mg^2+^ is incomplete. Increasing the dosage can improve this to some extent. Further increasing the dosage will form an NH_3_-NH_4_^+^ buffer system, keeping the solution pH essentially unchanged, making it difficult for Mg^2+^ to continue precipitating [22,23]. Therefore, the optimal NH_3_H_2_O/Mg^2+^ molar ratio is 9:1.

Within the range of Mg^2+^ concentration from 0.1 to 0.7 mol/L, the precipitation rate of Mg^2+^ increased rapidly with the increase in Mg^2+^ concentration (Fig 3b). When the concentration of Mg^2+^ in the solution is very low, the Mg²⁺ in the solution is not sufficient to combine with OH⁻ to form SSMH, resulting in a low precipitation rate of Mg² ⁺ . The excessive amount of Mg² ⁺ causes a sharp increase in the supersaturation of the solution, leading to a rapid and intense precipitation reaction. As the Mg^2+^ concentration reaches a sufficient level, between 0.7 and 1.3 mol/L, the precipitation rate of Mg^2+^ remains relatively constant. In this experiment, the Mg^2+^ concentration was determined to be 0.7 mol/L. The reaction temperature has a certain influence on the precipitation rate of Mg^2+^. At a low temperature of 30 °C, the precipitation rate of Mg^2+^ is relatively low at 72.5%. When the temperature is increased to 60 °C, the precipitation rate reaches its highest value of 85.5%. Further increasing the temperature leads to a slight decrease in the recovery rate (Fig 3c). This is because ammonia water does not completely ionize in water. When the temperature is raised, the ionization of ammonia water is promoted, enhancing its alkalinity and thereby increasing the recovery rate of Mg^2+^. However, at excessively high temperatures, ammonia gas tends to volatilize and be lost, resulting in a decrease in the recovery rate of Mg^2+^. In this experiment, the reaction temperature was determined to be 60 °C. When the reaction time was short, at 0.2 hours, the precipitation rate of Mg^2+^ was relatively low, at 67.7%. It reached 85.5% at 1 hour, and further extending the reaction time did not significantly change the recovery rate (Fig 3d). After the addition of ammonia water, Mg^2+^ react with the hydroxide ions produced by the ionization of ammonia to first form a supersaturated solution, then nucleate, and finally grow into SSMH precipitates. The nucleation of SSMH is a relatively slow process. Once the SSMH nuclei are formed, Mg^2+^ and hydroxide ions in the solution continuously grow around the nuclei until the product of their concentrations is less than or equal to the solubility product constant, at which point the precipitation rate of Mg^2+^ reaches its maximum [24]. In this experiment, the reaction time was determined to be 1 hour.

### 3.3 Effects of ammonia cycle times

**Fig 4:**
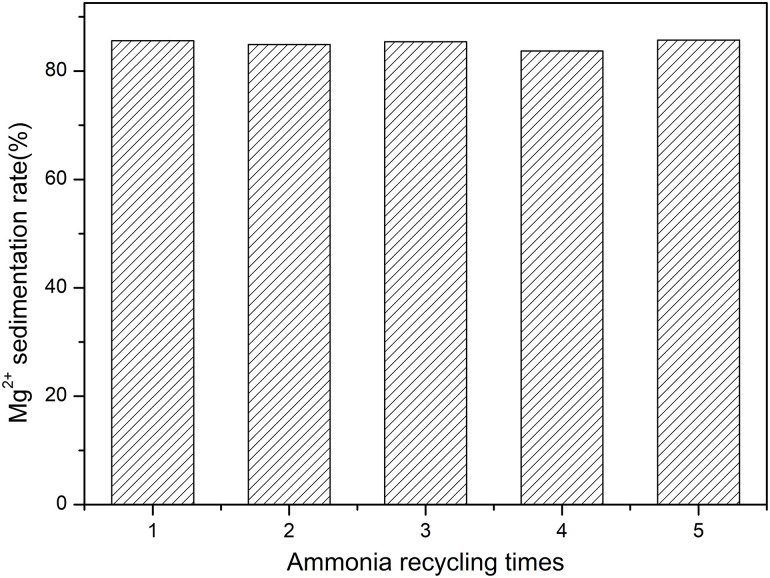
Effects of ammonia cycle times on Mg^2^^+^ precipitation rate.

The effects of ammonia cycle times on the precipitation rate of Mg^2+^ and RRX dye removal rate are shown in Fig 4.

From Fig 4, it can be observed that as the number of ammonia recycling times increases, the sedimentation rate of Mg² ⁺ remains at about 85%, showing a stable trend. Within the range of ammonia recycling times studied, the sedimentation rate of Mg² ⁺ does not significantly change with the increase in the number of recycling times. This result indicates that the preparation of SSMH using the ammonia recycling method is feasible, and the yield of SSMH is stable without significant changes. At the same time, the process of preparing SSMH reduces ammonia pollution and saves chemical reagents, making it an environmentally friendly scheme for synthesizing SSMH.

### 3.4 Characterization analysis of SSMH

#### 3.4.1 SEM analysis.

**Fig 5:**
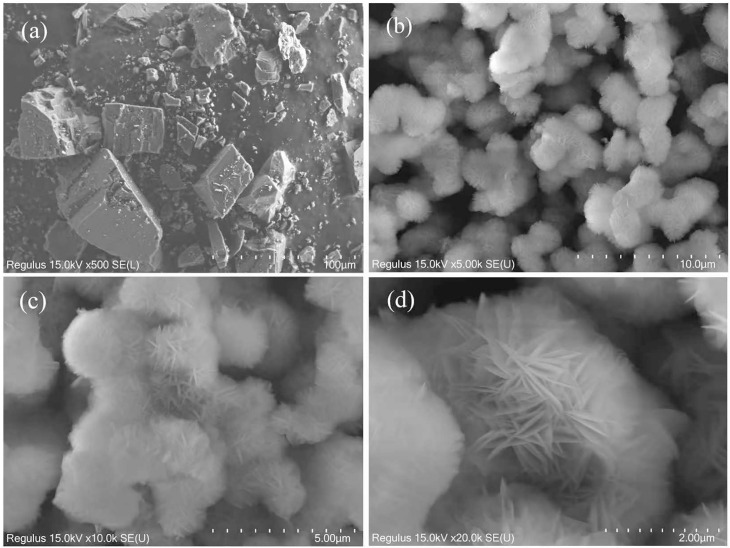
Morphological observation of synthesized SSMH. (a)magnesite, 500 × ; (b)synthesized SSMH, 5000 × ; (c)synthesized SSMH, 10000 × ; (d)synthesized SSMH, 20000 × .

SEM is a high-resolution microscopy technique primarily used to observe and analyze the surface morphology and microstructure of materials. Fig 5a depicts the unscreened magnesite powder after crushing. The particles exhibit irregular shapes, with some edges being relatively sharp while others are smoother. The surfaces of the particles are relatively smooth, and there is no aggregation between the particles. Fig 5b, 5c and 5d show images of the synthesized product, magnified 5000 times, 10000 times, and 20000 times, respectively. It presents a spike spherical shape, denoted as SSMH. These particles of SSMH are uniform in size, with a diameter of approximately 2 micrometers, which categorizes them as micrometer-sized materials. The morphology and structure of SSMH are different from those reported by Li et al [25]. Li et al used the ammonia recycling method, with industrial calcined powder as the raw material and ammonium salt solution as the recycling mother liquor, to synthesize layered magnesium hydroxide. Scanning electron microscopy characterization revealed that the magnesium hydroxide exhibited hexagonal flake shapes, with an average particle size of approximately 0.86 micrometers and nanoscale thickness, and it had good filterability.

The image shows that the SSMH particles are uniform with a small amount of agglomeration. The SSMH has a spike-like structure, which can significantly increase the specific surface area of the material. A high specific surface area means there is more surface area available for the adsorption of dye molecules, thereby improving the adsorption efficiency. The spike-like structure encloses many pores, providing more adsorption sites and channels, making it easier for dye molecules to come into contact with the inner surface of the adsorbent. The sharp edges and tips have a higher surface energy, making them more likely to interact with dye molecules and enhance the adsorption capacity. The larger size and specific shape of SSMH make it easy to separate from the aqueous solution. The sharp shape of SSMH helps to increase the contact area and rate of the reaction, improving its dye removal efficiency.

#### 3.4.2 Specific surface area analysis.

To quantify the adsorption performance of SSMH, a specific surface area analyzer is used to precisely measure its specific surface area and assess its adsorption capabilities. The pore volume can be estimated from the brunauer Emmett teller (BET) curve, a larger pore volume generally indicates better adsorption performance.

**Fig 6:**
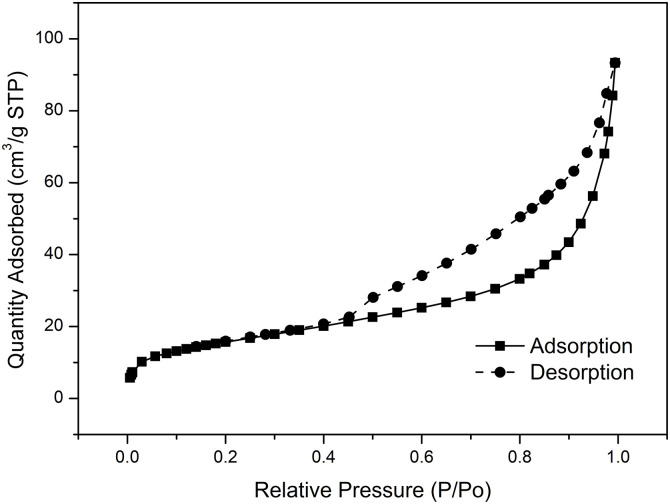
The N_2_ adsorption-desorption isotherm curve of synthesized SSMH.

According to International Union of Pure and Applied Chemistry (IUPAC) classification, N_2_ adsorption-desorption isotherms can be divided into six types (I-VI). Among them, Types II and IV are common for mesoporous and macroporous materials, and Type IV shows a clear hysteresis loop, which indicates the presence of mesopores or macropores. From Fig 6, it can be observed that the N₂ adsorption-desorption curve of SSMH exhibits a clear hysteresis loop, which consistent with the Type IV isotherm model. This indicates that SSMH has typical mesoporous and macroporous structures. The slight N₂ adsorption increase at the beginning of the curve (at low P/P₀ values) suggests the presence of a small amount of micropores. As P/P₀ increases (0.45–0.95), the significant N₂ adsorption increase confirms the development of mesopores.

As shown in Table 1, the specific surface area of SSMH is 56.8457 m²/g, which is higher than the specific surface area of 8 m²/g for magnesium hydroxide synthesized by Li et al [25]. The average pore diameter of SSMH is 10.1565 nm. In combination with the N₂ adsorption-desorption isotherms, it can be concluded that SSMH has a large number of mesopores. This pore size range allows dye molecules to easily enter the interior of the pores and adsorb onto the pore walls. Compared to microporous materials, mesoporous materials have larger pores, enabling dye molecules to diffuse more freely into the pores, thereby increasing the adsorption rate. Mesoporous materials often possess a high specific surface area, which means they have more surface area available for dye molecules to adsorb. Because the pore structure of mesoporous materials facilitates the diffusion of dye molecules, the adsorption process usually has faster kinetic characteristics. This means that mesoporous materials can reach adsorption equilibrium in a shorter time, improving treatment efficiency. Mesoporous materials can typically regain their adsorption capacity through simple heating or solvent washing, allowing for multiple cycles of use. This not only reduces treatment costs but also enhances the sustainability of the materials.

**Table 1:** Pore size data of synthesized SSMH.

specific surface area (m^2^/g)	average pore diameter (nm)	total pore volume (cm^3^/g)
56.8457	10.1565	0.0680

### 3.5 Optimization for treating RRX dye solution with SSMH

**Fig 7:**
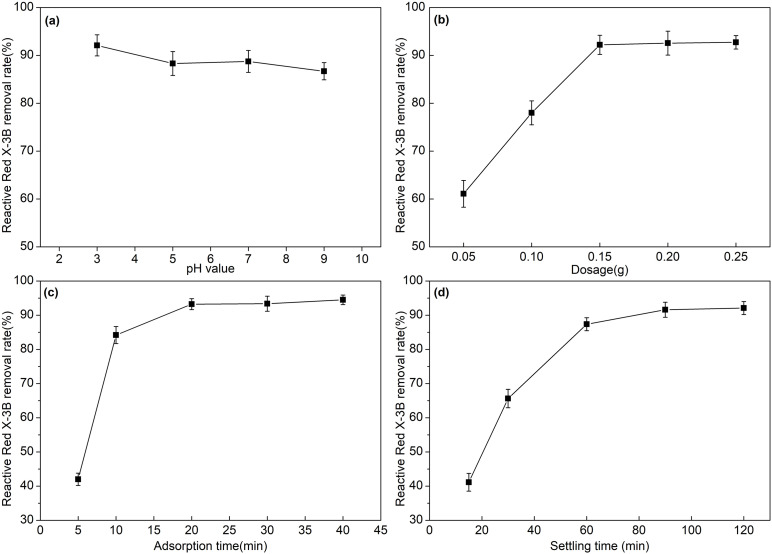
Effect of reaction conditions on RRX dye removal efficiency. (a)The effect of pH on the removal efficiency of RRX dye; (b)The effect of SSMH dosage on the removal efficiency of RRX dye; (c)The effect of adsorption time on the removal efficiency of RRX dye; (d)The effect of sedimentation time on the removal efficiency of RRX dye.

The RRX removal rate is slightly higher under acidic and neutral conditions compared to alkaline conditions (Fig 7a). Under acidic and neutral conditions, the OH⁻ concentration on SSMH surface is higher, which can engage in effective chemical reactions or electrostatic interactions with RRX molecules, and enhances the adsorption capacity. Additionally, RRX presents as a negative ion in acidic environments, and this form is more readily adsorbed by the positively charged SSMH surface [14,17]. As SSMH dosage increases, the RRX removal rate increases (Fig 7b). After the dosage reaches 0.15 g, the RRX removal rate changes are not significant. The initial RRX concentration and volume is fixed, and thus the number of dye molecules is constant, so, SSMH increase corresponds to an active adsorption sites increase, which enhances the RRX removal rate. However, as more SSMH adsorbent is added, the RRX dye, solution, and SSMH adsorbent reach a dynamic adsorption equilibrium. Even with additional adsorbent, the RRX removal rate no longer increases [17]. As adsorption time increases, the RRX removal rate increases (Fig 7c). The RRX removal rate is 94.5% at 20 minutes, which indicates that SSMH has a rapid adsorption reaction towards RRX. Under alkaline conditions, the surface of SSMH is typically positively charged, so it can adsorb anionic dyes like RRX. Alternatively, the active sites on SSMH may form complexes with the functional groups of RRX dye molecules, or chemical reactions between SSMH and RRX dye molecules may occur, and form insoluble precipitates. In summary, the mechanism of RRX adsorption by SSMH is a complex, multi-faceted process that involves electrostatic adsorption, surface complexation, precipitation reactions, and physical adsorption. As the active sites become saturated, the RRX removal rate gradually reaches equilibrium [17]. As the settling time increases, the RRX removal rate improves (Fig 7d). Many studies measure the concentration of dyes in the filtrate after filtering with membranes or filter paper, which can introduce experimental errors. In this experiment, we adopted the method of natural sedimentation and directly measured the concentration of dyes in the supernatant, which yields more accurate results. We found that SSMH, which adsorbs dyes, settles quickly. After sedimentation for 60–90 minutes, the removal efficiency of RRX dye reached 87.36–91.6%. The fast sedimentation is related to the relatively large particle size of SSMH [25]. Fast-settling adsorbents can rapidly separate from the solution, reducing processing time and enhancing efficiency. This is particularly significant for large-scale wastewater treatment, as it can substantially shorten the treatment cycle.

### 3.6 Adsorption isotherms

The adsorption isotherm model describes the interaction between the adsorbent and the adsorbate. Langmuir and Freundlich’s isotherm model was used to evaluate the data rather than adsorption. The adsorption isotherm determines the type of adsorption that occurs by analyzing the magnitude of the linear regression value generated by the graph on each isotherm so that the characteristics of the adsorption system between the solution and the adsorbent surface can be identified [14]. In this experiment, the study of adsorption isotherms was produced by varying the initial concentration of RRX dye aqueous solutions at obtained optimum pH and contact time. After reaching adsorption equilibrium with SSMH, the RRX concentrations in the solution were measured. The equilibrium adsorption capacity was calculated, and a scatter plot of equilibrium adsorption capacity versus equilibrium concentration was created.

**Fig 8:**
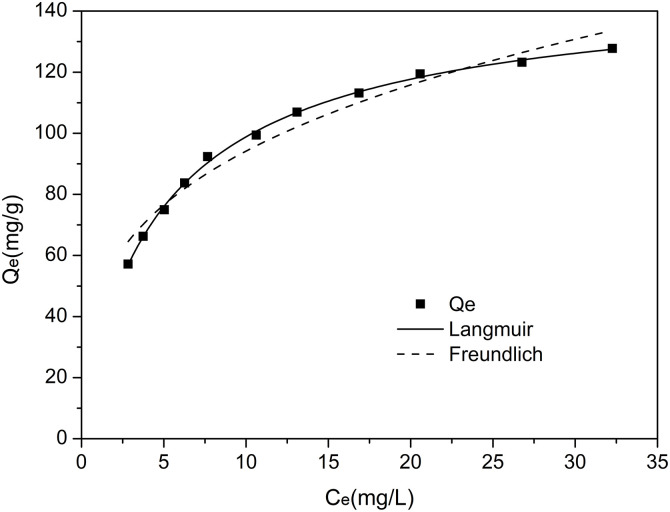
Adsorption isotherm model curves of SSMH.

Based on equations (2) and (3), the nonlinear curve of the Langmuir and Freundlich isotherm model on the adsorption of RRX dye using SSMH adsorbent is presented in Fig 8. Based on the values in Fig 8, the adsorption isotherm parameter values can be seen in Table 2. The data from Tabel 2 shows that the correlation coefficient R^2^ for the Langmuir equation is higher than the Freundlich model. This indicates that the adsorption pattern occurring on SSMH with RRX dye is monolayer with the Langmuir adsorption isotherm model.

**Table 2:** Fitting parameters for the isothermal RRX adsorption by SSMH.

models	parameters	numbers
Langmuir	Q_*m*_/(mg/g)	150.4944 ± 3.9610
	K_*L*_/(L/mg)	0.2367 ± 0.0088
	R^2^	0.998
Freundlich	K_*F*_/(mg/g)	47.3138 ± 2.6238
	n	0.2989 ± 0.020
	R^2^	0.9607

As shown in Table 2, the adsorption capacities of SSMH was 150.4944 mg/g at 25°C. Q_m_ is used for evaluating the adsorbents’ performances and plays an important role in screening suitable adsorbents. In this study, the adsorbates and adsorption temperature conditions were chosen to compare performance and efficiency. The Q_m_ values for the adsorption of Reactive red dye from different materials and other dyes from magnesium hydroxide reported elsewhere are presented in Table 3. This indicates that SSMH prepared by the ammonia recycling method have a satisfactory Reactive red dye removal performance compared to the various adsorbents previously reported.

**Table 3:** The Q_m_ of Reactive red dye on different materials vs. different dyes on magnesium hydroxide.

Dyes	Adsorbent	Q_m_ (mg/g)	Temperature (°C)	References
Reactive red 239	Cetyltrimethylammonium bromide-modified zeolite	15.9	35	[4]
Reactive red	Coconut tree flower carbon	181.9	35	[5]
Reactive red 120	cetylpyridinium modified Resadiye bentonite	81.97	25	[6]
Reactive Red 141	sodium hypochlorite-modified chitin	124	30	[7]
Reactive Red 194	Brazilian pine-fruit shell	20.8	25	[8]
Reactive Red MF-3B	sonication-surfactant-modified attapulgite clay	94.34	70	[9]
Reactive Red 198	*Aspergillus parasiticus* *fungal* biosorbent	0.1	35	[10]
Reactive Red 4	coke waste	70.3	30	[11]
Reactive Red dye	polyvinyl alcohol/sodium alginate copper-doped zinc oxide nanocomposite	67.98	25	[12]
Reactive Red X-3B	Magnesium hydroxide	150.49	25	This study
Congo red	Magnesium hydroxide	116.4	30	[13]
Congo red	Magnesium hydroxide	46.3	35	[14]
alizarin red S	Magnesium hydroxide	231.78	25	[15]
Congo Red	Magnesium Aluminium Hydroxide	52.3	25	[16]
Indigo Carmine	Magnesium Hydroxide	43.5	25	[17]

### 3.7 Adsorption kinetics

Adsorption kinetics aims to understand and investigate the mechanism of the process that controls the rate of adsorption. It is used for the selection of optimum operating conditions. The pseudo-first-order and pseudo-second-order are the most widely used models to study the adsorption kinetics of dyes and measure the rate of adsorption [14].

**Fig 9:**
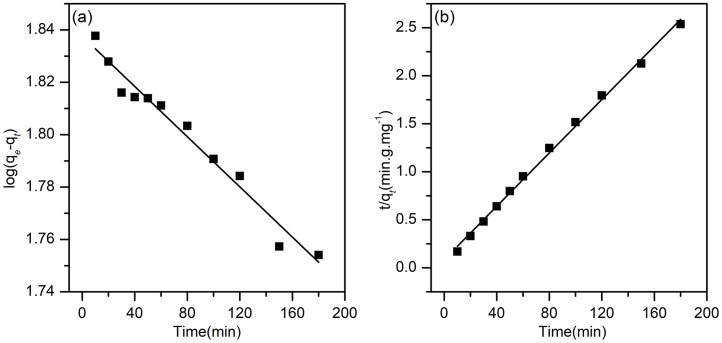
Pseudo-first-order fitting curve(a) and pseudo-second-order fitting curve(b) for the RRX adsorption by SSMH.

In this experiment, 80 mg/L RRX simulated wastewater was used. The RRX concentration in the solution was measured at different adsorption times to calculate the adsorption amount at time t. Based on equations (4) and (5), the linear curve of the pseudo-first-order and pseudo-second-order model on the adsorption of RRX dye using SSMH adsorbent is presented in Fig 9. Based on the values in Fig 9, the adsorption kinetics parameter values can be seen in Table 4.

**Table 4:** Kinetic fitting parameters for RRX adsorption by SSMH.

models	parameters	numbers
pseudo-first-order	K_1_(min^-1^)	0.0012
	R^2^	0.9709
	Q_e_(mg/g)	68.802
pseudo-second-order	K_2_(g ⋅ mg^-1^ ⋅ min^-1^)	0.0023
	R^2^	0.9973
	Q_e_(mg/g)	71.942

Based on the analysis of the adsorption kinetics data in Table 4, it is known that the adsorption kinetics model suitable for showing the RRX dye adsorption process is ideal for the pseudo-second-order model with a correlation coefficient (R^2^) of 0.9973. Similar results were found that the Congo Red dye adsorption was best explained by a pseudo-second-order model [14]. The value of R^2^ in pseudo-second-order shows that the adsorption occurs chemically, forming chemical bonds between the adsorbate molecule and the adsorbent surface.

### 3.8 Possible adsorption mechanism

**Fig 10:**
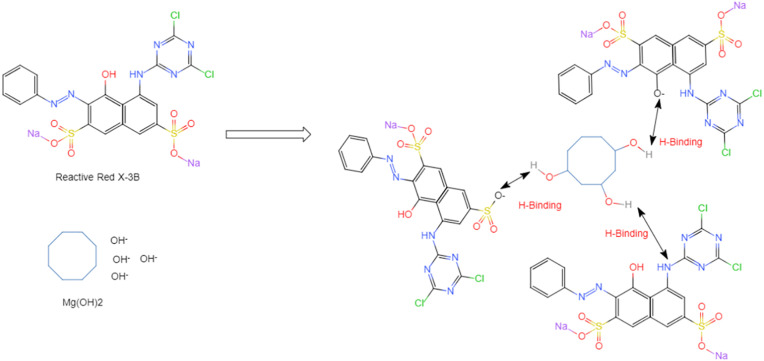
Possible mechanism of SSMH adsorption of RRX.

The adsorption of RRX dye by SSMH is related to the molecular structure of the dye. The possible mechanism is shown in Fig 10. The RRX dye molecule contain hydroxyl groups, amino groups, and oxygen anions. The hydrogen atoms of the amino functional groups, as well as the oxygen atoms of the hydroxyl groups and the oxygen anions in the dyes, form hydrogen bonds with the hydroxyl oxygen atoms in SSMH. These bonds are believed to play a significant role in the adsorption mechanism of SSMH for RRX.

The benzene ring of the RRX dye molecule contains OH groups. Due to its unique molecular structure, the OH groups in the RRX molecule exhibit weak acidity and can ionize hydrogen ions when RRX is in an aqueous solution. However, when the pH of the RRX solution varies between 3 and 9, there is no significant change in the adsorption capacity. This is because the hydroxide ions ionized from SSMH adjust the pH of the solution. Therefore, the OH groups in the RRX molecules first react with the free hydroxide ions in the solution. Subsequently, the RRX dye is strongly adsorbed onto the surface of SSMH through the hydrogen bonds formed between the negatively charged oxygen atoms in the RRX dye molecules and the hydroxyl oxygen atoms in SSMH, as shown in Fig 10.

## 4 Conclusions

This study has developed an innovative and environmentally friendly synthesis process for spike spherical magnesium hydroxide (SSMH), utilizing magnesite and an ammonia-cycle method to effectively eliminate waste liquid, gas, and chemical reagent pollution. The process involves calcining magnesite to obtain calcined powder, which then reacts with (NH_4_)_2_SO_4_ to produce magnesium sulfate and ammonia. Subsequently, magnesium sulfate reacts with ammonia water to generate SSMH. Under optimized conditions, the extraction rate of Mg² ⁺ can reach 93.4%, and the precipitation rate stabilizes at 85%. SEM confirms the spike spherical structure of SSMH, with uniform particle sizes of 2 μm. Adsorption studies show that SSMH has a maximum adsorption capacity of 150.4944 mg/g for Reactive Red X-3B dye at 25°C, driven primarily by chemisorption and fitting well with the Langmuir and pseudo-second-order kinetic models. In conclusion, this process is not only environmentally sustainable but also demonstrates broad application potential for SSMH in treating RRX aqueous solutions and removing heavy metals, persistent organic compounds, nitrogen, and phosphorus from various polluted sources.

## Supporting information

S1 File Supporting Information. (ZIP)
